# The Effects of Antisense miRNA-20a Alone or in Combination with Imatinib on K562 Cell Proliferation

**DOI:** 10.3389/fphar.2017.00127

**Published:** 2017-03-17

**Authors:** Ying Zhou, Dongmei He, Jinrong Zeng, Shijie Bao, Jing Lai, Yujun Weng, Shengting Chen

**Affiliations:** ^1^Departmemt of Hematology, The First Affiliated Hospital, Jinan UniversityGuangzhou, China; ^2^School of Medicine, Institute of Hematology, Jinan UniversityGuangzhou, China; ^3^Department of Internal Medicine, Guangzhou Nansha Central HospitalGuangzhou, China

**Keywords:** microRNA-20a, antisense oligonucleotide chain, K562 cells, imatinib, proliferation, cell apoptosis

## Abstract

**Objective:** The effects of microRNA-20a (miR-20a) antisense oligonucleotides (ASODNs) on the proliferation and apoptosis of K562 cells were investigated, and the effects of these ASODNs in combination with imatinib on K562 cells were preliminarily observed.

**Methods:** miR-20a ASODNs and scrambled oligonucleotides (SODNs) were chemically synthesized, and the later was used as the control. miR-20a ASODNs were transfected into K562 cells using Lipofectamine 2000 transfection reagent, and the expression of miR-20a was detected using real-time quantitative RT-PCR (qRT-PCR). The CCK8 assay was performed to detect the inhibition of the cell growth rate. The cells were stained by Hoechst 33258 to detect apoptotic cell morphology. Annexin V/PI double staining was used to detect the cell apoptosis rate using flow cytometry. The protein expression levels of E2F1, P21, and Bim in the K562 cell line were detected using western blotting.

**Results:** The qRT-PCR results showed that the expression level of miR-20a in K562 cells transfected with miR-20a ASODNs was lower than those in the normal control, SODN and blank transfection groups (*p* < 0.05). miR-20a ASODNs significantly inhibited the growth of K562 cells as compared to the controls (*p* < 0.05). The Hoechst staining results showed morphological changes, suggesting apoptosis. The cell apoptosis rates in the ASODN group was (13.9 ± 1.5)%, which was significantly higher than that in the normal control group (1.84 ± 0.21)%, blank transfection group (3.21 ± 0.32)%, and SODN group (3.72 ± 0.44)% (*p* < 0.05). The protein expression of E2F1 and P21 in K562 cells transfected with miR-20a ASODNs were higher, while the level of Bim protein was significantly lower than that in the control groups. When miR-20a ASODNs were combined with imatinib, the growth of K562 cells was significantly inhibited as compared to the ASODN treatment alone, imatinib alone, and SODN+imatinib groups (*p* < 0.05).

**Conclusions:** miR-20a ASODNs could induce apoptosis and inhibit the proliferation of K562 cells. In addition, imatinib combined with miR-20a ASODNs can increase the inhibitory effect on K562 cell proliferation.

## Introduction

microRNAs (miRNAs) comprise a group of non-coding small molecule RNAs. miRNA genes account for 1% of the total human genome (Shao-Yao, 2007), yet they play important regulatory roles in the physiological activities of cells, such as growth, differentiation, proliferation, and apoptosis. Accumulative literature data suggest that the abnormal expression and regulation of miRNA are involved in the development and progression of many types of tumors (Croce, 2009). In addition, Analysis of miRNA expression profiles in chronic myeloid leukemia (CML) patients and healthy controls showed that the abnormal expression of some miRNAs was closely associated with the abnormal proliferation of CML cells (Venturini et al., 2007; Zhu et al., 2014). Therefore, miRNAs are new targets for the diagnosis and treatment of cancers, and open new avenues for cancer therapy.

The miR-17-92 cluster genes are located in the third intron of the C13orf25 gene on 13q31 and include miR-17-5p, miR-17-3p, miR-18, miR-19a, miR-19b, miR-20a, and miR-92-1 isoforms. They are closely associated with tumor development and are highly expressed in solid tumors and malignant hematologic disease (He et al., 2005; Fassina et al., 2012; Jin et al., 2013; Scherr et al., 2014). It has been shown that the expression of miR-17-92 cluster genes are increased in CD34^+^ cells in the early chronic phase of CML through the *BCR-ABL-c-MYC*-miR-17-92 pathway (Venturini et al., 2007). miR-20a belongs to the miR-17-92 family and is abnormally expressed in various tumors, such as lung cancer, gastric cancer, and colorectal cancer (Zhang et al., 2014; Fuziwara and Kimura, 2015; da Silva Oliveira et al., 2016). The high miR-20a expression can attenuate the expression of PTEN to promote tumor development (Fuziwara and Kimura, 2015). miR-20a is closely associated with the growth, invasion, and metastasis of tumor cells (Huang et al., 2012; Qiang et al., 2014; Zhang et al., 2014; Wang et al., 2015; Zhao et al., 2015). The study by Zhang et al. (2014) showed that the expression of miR-20a increased in colorectal cancer and was closely associated with the lymph node and distant metastases. In addition, high miR-20a expression could induce tumor cell resistance to chemotherapeutic drugs through different target genes (Chai et al., 2011; Zhu et al., 2016). Therefore, downregulation of miR-20a may increase the sensitivity of tumor cells to chemotherapeutic drugs.

Imatinib is a drug that specifically inhibits tyrosine kinase activity in the CML fusion gene, BCR/ABL1. However, studies have increasingly shown that drug insensitivity or drug resistance occurs during treatment of CML using imatinib. It is not clear whether downregulation of miR-20a expression can enhance the inhibitory effect of imatinib on K562 cell proliferation. Therefore, this study aimed to study the effects of miR-20a downregulation on K562 cell proliferation and apoptosis and changes in the expression of its related target proteins. In addition, the effects of the combination of miR-20a and imatinib on K562 cell proliferation were further investigated to provide new insight for targeted treatment of CML.

## Materials and methods

### Materials

The miRNA-20a antisense oligonucleotide (ASODN): 5′GUACCUGCACUAUAAGCACUUUA-3′ and the scrambled sequence: 5′CAGUACUUUUGUGUAGUACAA-3′ were synthesized by Shanghai GeneChem Co., Ltd., (China). Gibco RPMI 1640 culture medium was purchased from HyClone (USA). Lipofectamine 2000 Reagent was purchased from Invitrogen (USA). Imatinib mesylate was purchased from Selleck (USA). The CCK-8 Kit was purchased from ProbeGene Life Sciences (China). The fluorescence quantitative PCR reagent kit was purchased from RiboBio Co., Ltd., (China). Antibodies were purchased from Abcam (USA).

### Methods

#### Cell culture and transfection

K562 cells were maintained in RPMI-1640 culture medium containing 10% fetal bovine serum (FBS), 100 U/ml penicillin, and 100 U/ml streptomycin and were continuously cultured in an incubator with saturated humidity (37°C and 5% CO2). The medium was refreshed every other day. Cells at the logarithmic growth phase were used for experiments. Transfection was performed using the Lipofectamine 2000 transfection reagent according to the instruction manual. The experimental cells were divided into the miR-20a ASODN group, scrambled oligonucleotides (SODNs) group, simple liposome group (blank transfection group), and cell group (the blank group).

#### Detection of the relative expression level of miR-20a using fluorescence real-time quantitative PCR

Cells were collected after 48 h of transfection. RNA was extracted using the TRIzol reagent kit, and first strand cDNA was synthesized by reverse transcription using random primers and a reverse transcription reagent kit. The expression of miR-20a was detected using the SYBR Green I dye method with U6 as the internal control. The total reaction volume was 20 μL. The reaction condition was 95°C 10 min for pre-denaturation followed by 40 cycles of amplification. Each amplification cycle included 95°C 2 s, 60°C 30 s, and 70°C 10 s. The plate was read once at 70°C. Next, the fluorescence values were recorded every 5 s from 70 to 95°C at the changing rate of 0.5°C/s to obtain the melting curve. The relative expression levels in the cells were calculated using the relative quantitative formula: 2^−ΔCt^ × 100%, ΔCt = Ct(miR-20a)−Ct(U6).

#### Detection of cell proliferation using the CCK8 method

K562 cells at the logarithmic growth phase were inoculated onto 96-well plates, and transfection was performed according to the above procedures. At 48 and 72 h post transfection, 10 μL of CCK8 was added to each well and incubated at 37°C for 4 h. The absorbance (A450) at the wavelength of 450 nm were measured in a microplate reader. The A450 value reflected the number of surviving cells. After miR-20a ASODN transfection, cells were treated with combined 0.6 μmol/L imatinib and cultured in an incubator for 48 and 72 h. The A450 values of the cells in each group were measured, and the proliferation inhibition rates of the cells were estimated by the equation: The cell proliferation inhibition rate (%) = (1–A450 in the experimental group/A450 in the control group) × 100%. The experiments in each group were repeated three times.

#### Observation of apoptotic cell morphology using hoechst 33258 staining

After 72 h of transfection, cells were collected, centrifuged, and fixed in 0.5 ml of 4% formaldehyde solution for 10 min. The cells were then washed with PBS twice, the supernatant was discarded, and the remaining liquid was aspirated using a pipette. Cells were resuspended in 20 μL of DEPC water, evenly spread onto slides, and naturally dried. The Hoechst 33258 staining solution was evenly dropped onto slides, and staining proceeded for 5 min in the dark. The slides were washed with distilled water (ddH_2_O) twice, and excess water was soaked up to allow the slides to dry. The slides were covered with clean cover slips, and the stained cell morphology was observed under a fluorescence microscope.

#### Detection of cell apoptosis rates using annexin V/PI double staining and flow cytometry

Cells were centrifuged, collected, and washed with PBS twice. The supernatant was discarded, and the cells were resuspended in 400 μL of 1 × Binding Buffer and incubated with 5 μL of Annexin V-FITC for 15 min in the dark. Cells were mixed thoroughly with 10 μL of PI staining solution and incubated for 5 min in the dark. The cell apoptosis rate was detected using a flow cytometer.

#### Detection of the expression of target proteins using western blotting

K562 cells in each group were collected in 1.5-ml centrifuge tubes after 48 h of transfection. The total cellular protein was extracted using a protein extraction kit (Sigma,USA) and quantitated using the BCA method. The protein lysate (100 μl) was mixed with the sample loading buffer (1.0 M Tris-HCl (pH 6.8) 1.0 ml, 10% SDS 6.0 ml, β-mercaptoethanol 0.2 ml, and ddH_2_O 2.8 ml) and denatured at 100°C for 5 min. Proteins were separated in 10% SDS-PAGE and electrotransferred onto a nitrocellulose membrane. The membrane was incubated with primary antibodies (E2F1 1:200, P21 1:1,000–2,000, and Bim 1:500) at 4°C overnight, and GADPH primary antibody was used as the internal control. On the second day, the membrane was incubated with secondary antibodies [GVPDH (mouse anti-rabbit) 1:6,000, E2F1, P21, and Bim secondary antibodies 1:2,000] at room temperature for ~1 h on a shaker at 75 rpm. The secondary antibodies were washed out with TBST. Developing solution (Multi Sciences, China) was prepared at 1:1, and the membrane was placed in a UV gel image analyzer and photographed.

### Statistical analysis

SPSS13.0 software (SPSS Direct,Chicago) was used for the statistical analysis of the data. Experimental data are expressed as the mean ± standard deviation. The comparison of data between two groups was performed using a two-sample *t*-test. The comparison of data among multiple groups was performed using the analysis of variance of completely randomized design and random block design. The comparison of differences between groups was examined using the S-N-K test, which could perform pairwise comparisons of mean values of multiple samples. α = 0.05 was used as the significance level, and *P* < 0.05 indicated statistical significance.

## Results

### Detection of miR-20a expression in K562 cells after transfection using qRT-PCR

#### Detection of miR-20a expression using qRT-PCR

After two-fold serial dilution of cDNA from the K562 cell line, amplification of the target gene (*miR-20a*) and the internal control gene (*U6*) was performed to make relative standard curves. The correlation coefficients were 0.996 and 0.994 for U6 and miR-20a, respectively. After the PCR reaction, the melting curve peaks of U6 and miR-20a formed a specific single peak. The peak values of the melting curves of the miR-20a amplification products were all at 82.6°C, and the peak values of the melting curves of the U6 amplification products were all at 82.7°C. The amplification efficiencies of *miR-20a* and *U6* were between 95 and 100%, demonstrating the consistency of the amplification efficiencies for *miR-20a* and *U6*. Therefore, the relative quantitation of miR-20a expression could be calculated using the 2^−ΔCt^ × 100% formula.

#### miR-20a expression level in K562 cells after transfection with miR-20a ASODNs

**Figure 1:**
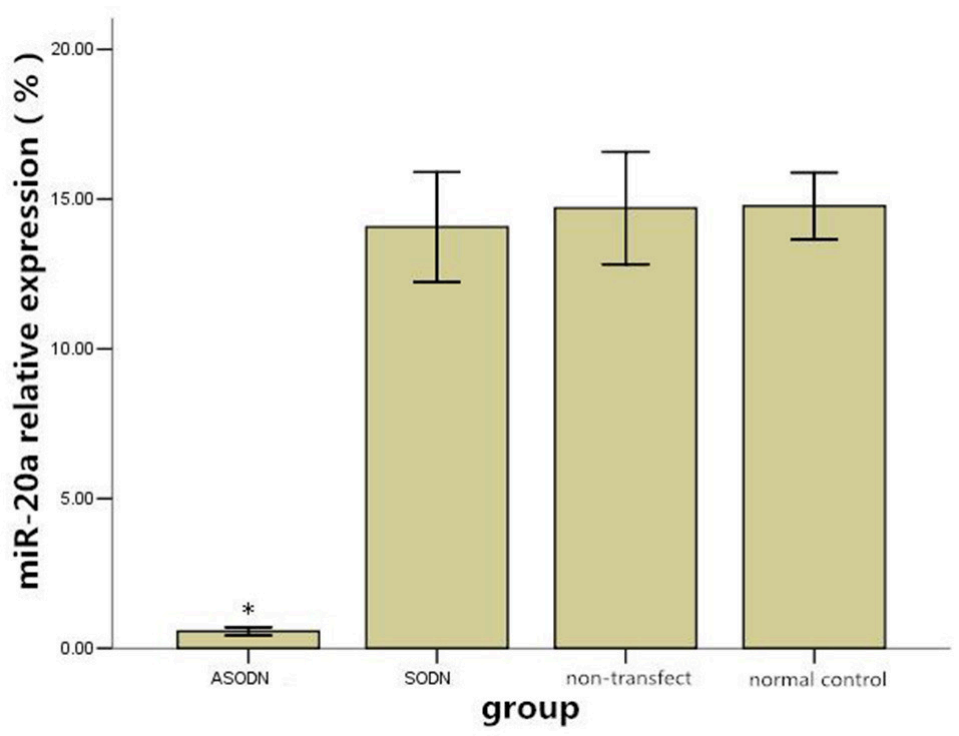
The effect of miR-20a ASODN transfection on the expression level of miR-20a in K562 cells (^*^P < 0.05).

After K562 cells were transfected with miR-20a ASODNs for 48 h, the relative expression level of miR-20a was calculated using the 2^−ΔCt^ × 100% formula (Figure 1). Compared to those in the blank control group, blank transfection group, and SODN group, the relative expression level in the ASODN group was significantly downregulated (*P* < 0.05). No significant changes of miR-20a expression were detected among the cell control group, blank transfection group, and SODN group (*P* > 0.05).

### K562 cell proliferation after transfection with miR-20a ASODNs

**Figure 2:**
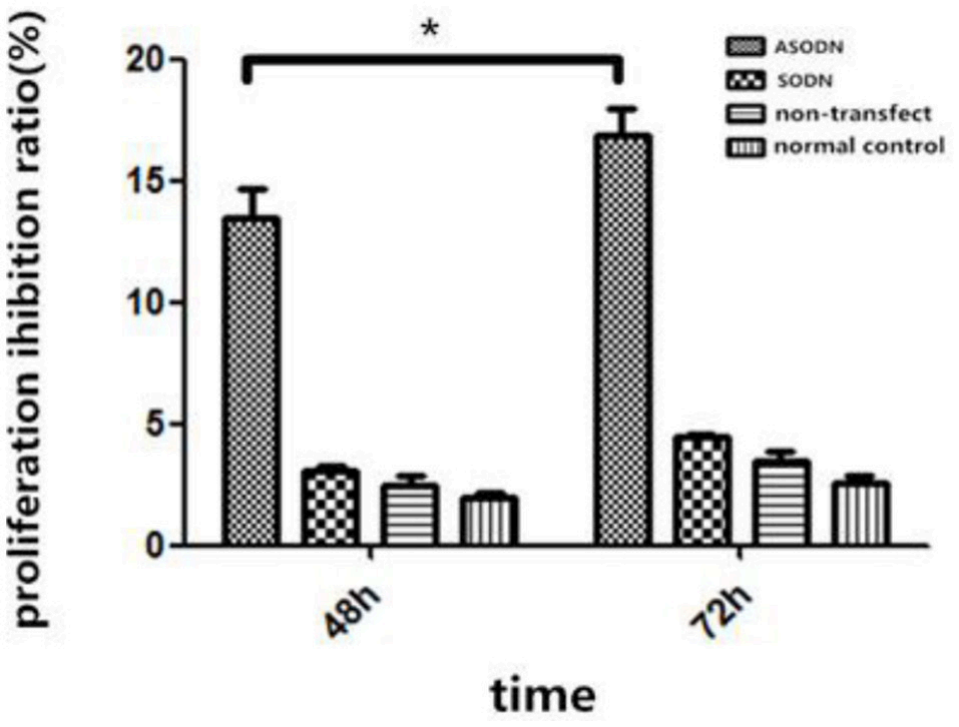
Inhibition of K562 cell growth by miR-20a ASODN (^*^P < 0.05).

K562 cells were transfected with miR-20a ASODNs, and the cell growth was detected using the CCK8 method (Figure 2). After transfection for 48 and 72 h, cell proliferation in the ASODN group was significantly inhibited, as compared to those in the normal control group, blank transfection group, and SODN group (Figure 2, *P* < 0.05). And, the difference of inhibition between the 48 and 72 h treatment in the ASODN group had statistical significance (*P* < 0.05). The cell control group, blank transfection group, and SODN group were not significantly different (*P* > 0.05).

### Detection of cell apoptosis morphology using hoechst staining

**Figure 3:**
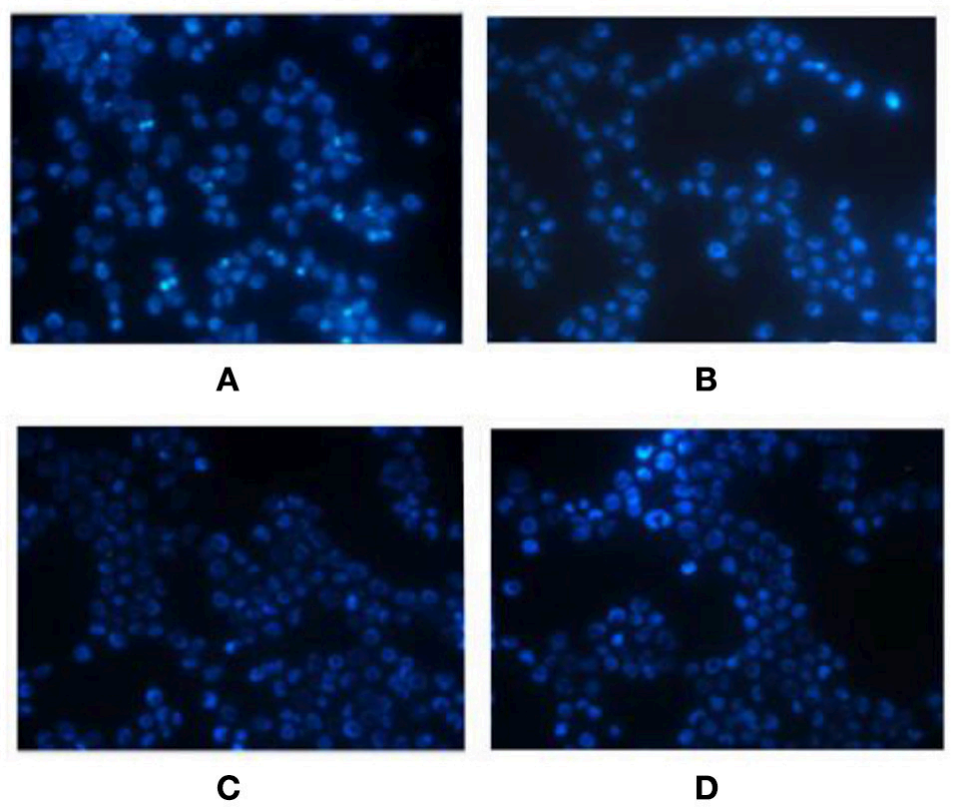
**Morphological changes of K562 cells after miR-20a ASODN transfection for 72 h detected by Hoechst fluorescence stain (×400)**. **(A)**, ASODN group; **(B)**, SODN group; **(C)**, Blank transfection group; **(D)**, Cell control group.

The Hoechst staining results showed that K562 cells exhibited apoptotic morphological changes after miR-20a ASODN transfection for 72 h. The K562 cells exhibited morphological changes of apoptosis including decreased cell volume, nuclear condensation, and nuclear fragmentation. The K562 cells in the cell control group, blank transfection group, and SODN group did not exhibit apoptotic morphological changes (Figure 3).

### Detection of apoptosis rates of K562 cells after miR-20a ASODN transfection using flow cytometry

**Figure 4:**
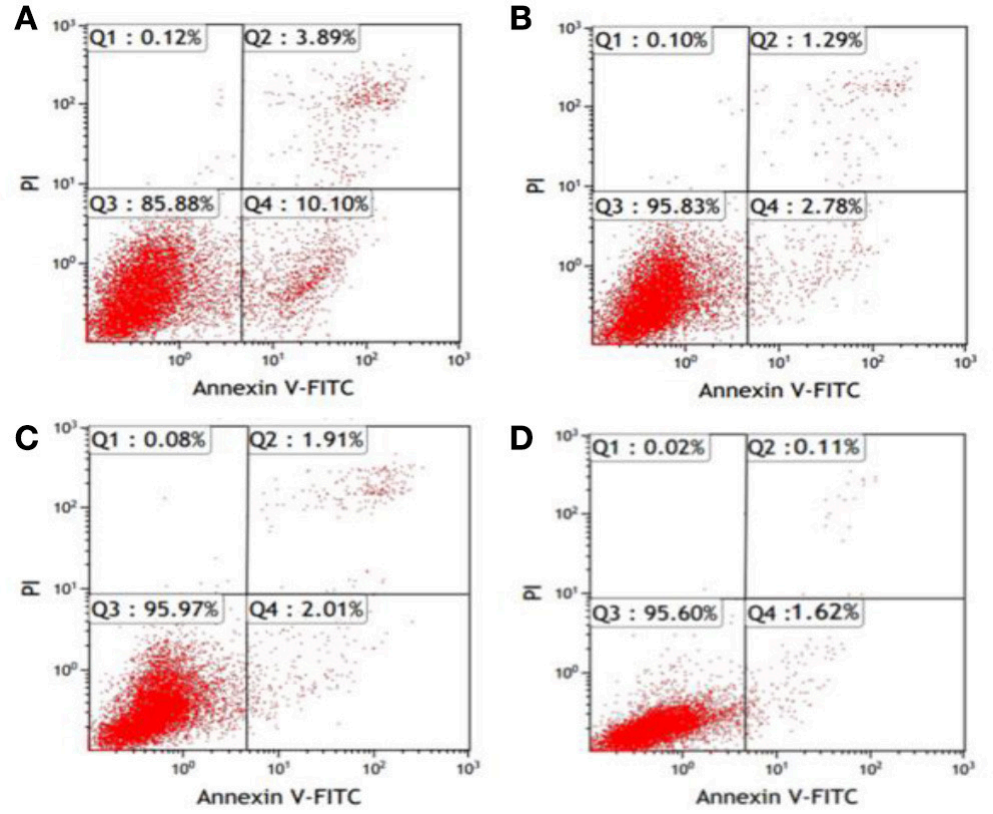
**Flow cytometric detection of K562 cell apoptosis after miR-20a ASODN transfection for 48 h**. (Annexin V/PI double staining method; the figure was a representative result). **(A)**, ASODN group; **(B)**, SODN group; **(C)**, Blank transfection group; **(D)**, Cell control group.

After K562 cells were transfected with miR-20a ASODNs for 48 h, the early-stage and late-stage apoptosis rates of cells were detected using the Annexin V/PI double staining method and flow cytometry. The results showed that the total apoptosis rate of cells in the ASODN group significantly increased, and the apoptosis rate was (13.86 ± 1.55)%, which was significantly higher than those in the blank control group, blank transfection group, and SODN group; the differences all had statistical significance (*P* < 0.05). The apoptosis rates in the latter three groups were (1.84 ± 0.21), (3.21 ± 0.32), and (3.72 ± 0.44)%, respectively, with no significant differences among the control groups (*P* > 0.05) (Figure 4).

### Detection of the expression levels of miR-20a target proteins using western blotting

**Figure 5:**
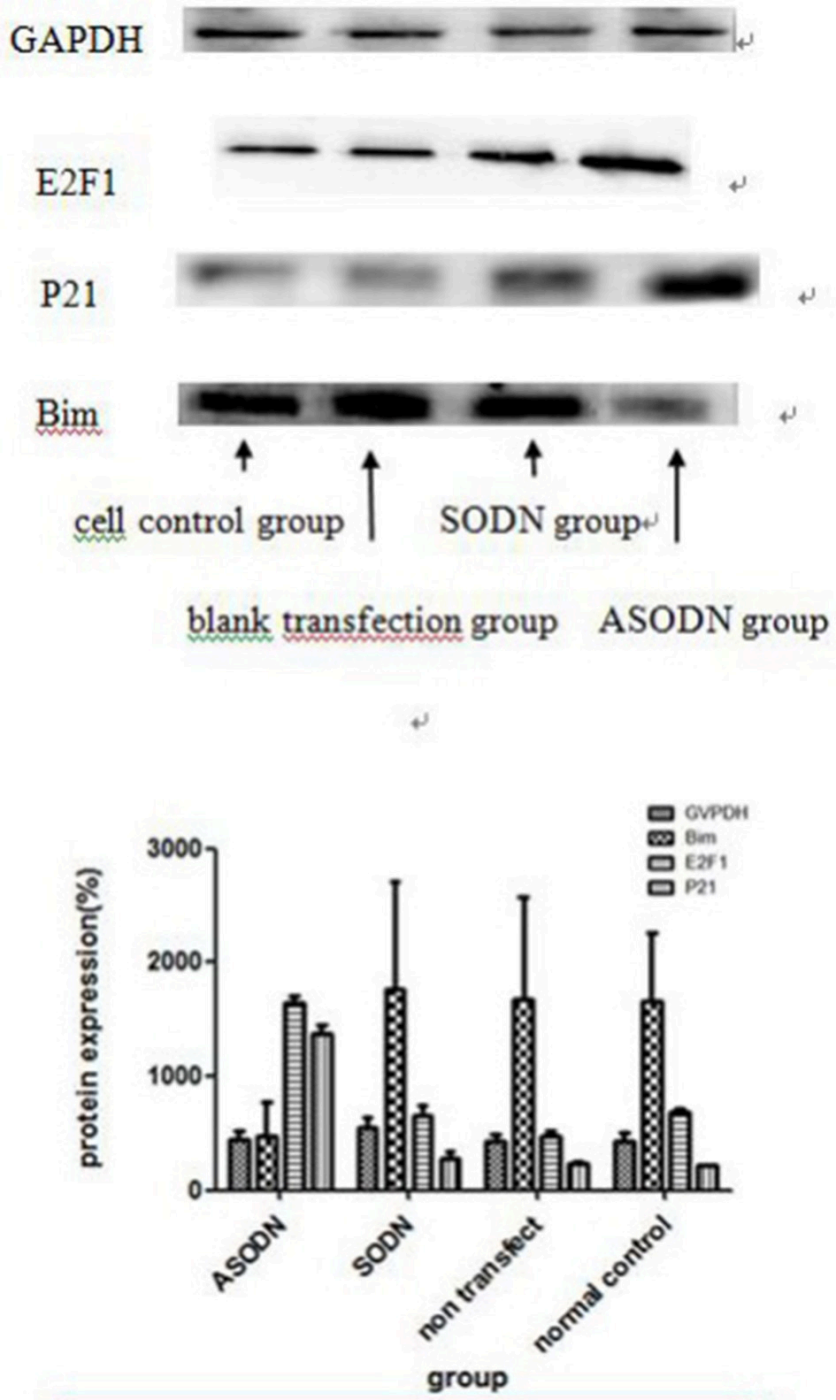
**The expression of miR-20a target proteins after K562 cells were transfected with miR-20a ASODNs for 48 h**.

After the K562 cell line was transfected with miR-20a ASODNs for 48 h, the expression levels of four proteins, the internal control protein GAPDH and the target proteins, E2F1, P21, and Bim, were detected using western blotting (Figure 5).The results showed that the expression levels of E2F1 and P21 proteins in the miR-20a ASODN group were higher than those in the cell control group, blank transfection group, and SODN group, while the expression level of Bim protein in the miR-20a ASODN group was lower than that in the other three groups. The expression level of the internal control protein was the same in all the groups.

### The inhibitory effect of miR-20a ASODNs combined with imatinib on the growth of K562 cells

**Figure 6:**
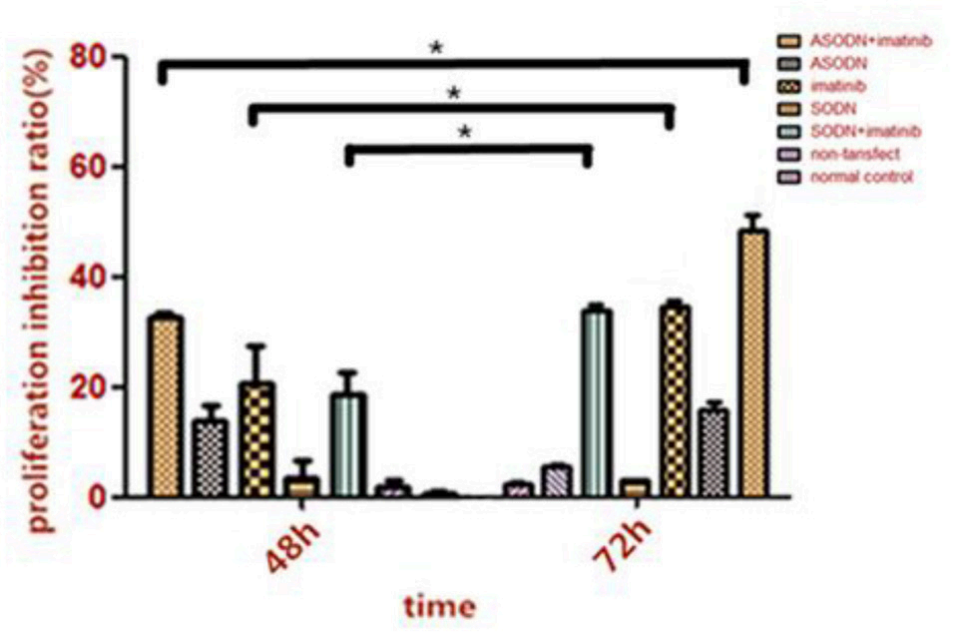
The inhibitory effect of miR-20a ASODNs combined with imatinib on the growth of K562 cells (^*^P < 0.05).

The cell growth rate of K562 cells transfected with miR-20a ASODN transfection was measured using the CCK8 method when cultured with 0.6 μmol/L imatinib (Figure 6). After transfection for 48 and 72 h, cell proliferation in the group with the combination of miR-20a ASODNs transfection and imatinib incubation was significantly suppressed; compared to that in the ASDON alone group, imatinib alone group, and SODN+imatinib group (*P* < 0.05). In addition, with increasing reaction time, the effect gradually increased. The comparison of the results between 48 and 72 h showed that the difference had statistical significance (*P* < 0.05). The experiments were repeated three times in each group.

## Discussion

It has been well-documented that miRNAs are involved in the development and progression of a variety of tumors, including hematologic tumors. The miR-17-92 cluster genes are abnormally amplified in a variety of tumors and have carcinogenesis in tumor cells (Hayashita et al., 2005; Fontana et al., 2008; Inomata et al., 2009). Venturini et al. (2007) showed that compared to normal CD34^+^ cells, CD34^+^ cells in the early chronic phase of CML had significantly higher expression levels of the miR-17-92 cluster genes. They also showed that *c-Myc* could regulate the expression of the miRNA-17-92 cluster genes, while *c-Myc* could be activated by the product of the *BCR-ABL* genes. In addition, they compared the expression of 210 miRNAs in K562 cells using the miRNA microarray method. The results showed that the expression of the mature miR-17-92 cluster genes decreased followed by the BCR-ABL kinase activity being suppressed by imatinib or the BCR-ABL expression being downregulated by RNAi. In addition, the inhibition of K562 cell proliferation and survival by downregulation of Myc by RNAi could be partially blocked by lentivirus-mediated overexpression of miR-17-19b-1, the other member of the miR-17-92 cluster family. Therefore, the miR-17-92 cluster family may be closely associated with CML. The results in this study showed that miR-20a, an isoform of miR-17-92 cluster genes was highly expressed in K562 cells.

In this study, after K562 cells were transfected with miR-20a ASODNs, the expression level of miR-20a significantly decreased, and K562 cell proliferation was significantly inhibited. In addition, the effects appeared to be time-dependent. Therefore, our results indicated that miR-20a ASODNs could specifically inhibit K562 cell proliferation. The Hoechst staining results showed that after K562 cells were transfected with miR-20a ASODNs, the cells exhibited morphological changes of apoptosis, such as decreased cell volume, nuclear condensation, and nuclear fragmentation. In addition, the apoptosis rate of K562 cells significantly increased after miR-20a ASODN transfection. Therefore, these results indicated that miR-20a ASODNs could promote K562 cell apoptosis, leading to the inhibition of cell proliferation. Matsubara et al. (2007) used ASODN to inhibit miR-17-5p and miR-20a, which caused apoptosis in lung cancer cells that expressed high levels of miR-17-92. The study of Dereani et al. (2014) also showed that ASODN targeting miR-17, the other member of the miR-17-92 cluster, could inhibit the growth of chronic lymphocytic leukemia (CLL) *in vivo* and *in vitro*. Our study results were consistent with the results reported in the above literature.

To investigate the molecular mechanism underlying the inhibition of K562 cell proliferation by miR-20a ASODNs, we further monitored the expression of the miR-17-92 target proteins *E2F1* (O'Donnell et al., 2005), *P21* (Inomata et al., 2009; Wong et al., 2010), and *Bim* (Mavrakis et al., 2010) using western blotting after the miR-20a in K562 cells was down-regulated by ASODNs. *E2F1* regulates the transition of cells from the early G_0_/G_1_ phase to the S phase and can promote cell apoptosis; therefore, it is an important cell cycle regulator. O'Donnell et al. (2005) showed that *c-Myc* regulated proliferation signals through the activation of *E2F1* transcription and regulation of its translation. *c-Myc* is a well-known transcription factor closely associated with the oncogenic function of the miRNA-17-92 family. Our results showed that after transfection with miR-20a ASODNs, the expression level of E2F1 protein in K562 cells significantly increased, suggesting that miR-20a in K562 cells may target *E2F1* gene expression regulation to promote cell proliferation.

As a negative regulator of the cell cycle, P21 is closely associated with the cell cycle, differentiation, aging, and apoptosis and mediates the activity of transcription factors. In an MLL leukemia animal model, the miR-17-92 cluster regulated the pathogenic ability of leukemia stem cells through regulating the expression of the cell cycle factor P21 (Wong et al., 2010). miR-17 and miR-20a, which have conserve high gene hemology in the miR-17-92 cluster, could promote the proliferation of CML cells and B cell lymphoma cells through targeted regulation of P21 expression (Inomata et al., 2009). The results in this study showed that the expression level of P21 protein in K562 cells in the ASODN significantly increased after miR-20 ASODN transfection, suggesting that miR-20a ASODNs induced K562 cell apoptosis. The inhibition of cell proliferation may be associated with the targeted regulation of P21 protein by miR-20a.

The *Bim* (Bcl-2 interacting mediator of cell death) gene is a tumor suppressor (Bouillet et al., 2001). Yan et al. showed that inhibition of miR-17-5p expression upregulated the protein expression level of its target gene, *Bim*, to increase the sensitivity of pancreatic cells to gemcitabine (Yan et al., 2012). This study showed that the expression level of Bim protein in K562 cells transfected with miRNA-20a ASODNs significantly decreased, which was not consistent with the expected results. This result suggested that *Bim* in K562 cells may not be directly regulated by miR-20a; further studies are warranted to elucidate the mechanism.

This study further investigated the effect of miR-20a ASODNs combined with imatinib on K562 cell proliferation. The CCK8 detection results showed that miR-20a ASODNs combined with imatinib significantly inhibited K562 cell growth as compared to the growth of the miR-20a ASODN alone, imatinib alone, and SODN+imatinib groups. Therefore, miR-20a ASODNs along with imatinib could increase the inhibitory effects on K562 cell proliferation. Lopotová et al. (2011) noted that tyrosine kinase inhibitors (TKIs) such as imatinib could inhibit *bcr-abl* expression to increase miR-451 expression. The destruction of the regulatory loop between *bcr-abl* and miR-451 may help to increase the treatment efficacy in CML patients. Liu et al. (2012) showed that after CML cells were transfected with miR-144/451, the apoptosis-inducing effect of imatinib was promoted to overcome drug resistance to imatinib. The above literature reports together with our findings demonstrated that miRNA expression changes were closely associated with the sensitivity of CML cells to imatinib.

Collectively, we concluded that miR-20a ASODNs can induce K562 cell apoptosis and inhibit K562 cell proliferation. In addition, miR-20a ASODN, in combination with imatinib, results in an enhanced inhibiting effect on the proliferation of K562 than miR-20a ASODN or imatinib alone. The function of miR-20a ASODNs in the inhibition of K562 cell proliferation may be associated with the up-regulation of the target proteins E2F1 and P21, which are regulated by miR-20a.

## Author contributions

SC and DH contributions to the design of the work. YZ, JZ, and SB performed research and analyzed data. JL and YW contributed vital reagents and analytical tools. YZ and SC wrote the paper.

### Conflict of interest statement

The authors declare that the research was conducted in the absence of any commercial or financial relationships that could be construed as a potential conflict of interest.
